# Procedure-Related Complication of Willis Covered Stent in the Treatment of Blood Blister-Like Aneurysm: Stent Detachment from Dilating Balloon

**DOI:** 10.3389/fneur.2017.00639

**Published:** 2017-11-30

**Authors:** Yuxiang Zhang, Yupeng Zhang, Fei Liang, Chuhan Jiang

**Affiliations:** ^1^Beijing Neurosurgical Institute, Beijing Tiantan Hospital, Capital Medical University, Beijing, China

**Keywords:** complication, blister-like aneurysm, Willis covered stent, stent detachment, tortuosity of the internal carotid artery

## Abstract

The use of Willis covered stent (WCS) for intracranial aneurysms has increased based on the promising results of previous studies about its safety and effectiveness. With the accumulation of cases, reports about peri-procedural complications are emerging. In our department, 25 patients were treated with WCS during December 2015 to March 2017. We here reported an unexpected technical complication occurred in the treatment with the WCS for a blood blister-like aneurysm (BBA). During the procedure, the distal end of the stents detached from the dilating balloon partially or as a whole. This was attributed to the tortuosity of the access route and the extracorporeal gas exhaust maneuver. Then we applied a half-dilating technique to retrieve the detached stent. The procedures were detailed in this report and the possible reasons and approaches to avoid it were explored.

## Introduction

Intracranial blood blister-like aneurysm (BBA) is a type of aneurysm that lacks both the intima and media, and tends to rupture. It remains a demanding task to find a safe and effective approach to eliminating this lesion because of the fragility of artery wall (1–3). Nowadays, there are several options to repair BBA, such as microsurgical clipping or wrapping, endovascular coiling with balloons or stents, flow diverter such as pipeline embolization device, and covered stents such as the WCS. Despite all these choices, overlapping flow diverters and the use of WCS are favored in our center and as well proved more efficacy in the literature (3, 4). Willis covered stent (WCS, MicroPort, Shanghai, China) has been used to treat intracranial aneurysms (AN) since the last decade and the outcome is favorable (5). Different from other covered stent such as Jostent (JCS, Abbott Vascular, IL, USA, GER), WCS enables us to treat AN distal to the C5 segment of internal carotid artery (ICA). However, due to that it is a covered stent in nature, the stiffness and the requirement of a balloon for expansion might introduce more procedure-related complications when compared with other self-expandable stents such as Neuroform (Neuroform stent, Stryker Neurovascular, Fremont, CA, USA). In our department, 25 patients was treated with WCS during December 2015 to March 2017, including 4 cases (16%) for carotid-cavernous fistula and 21 cases (84%) for AN. In two cases (8%), we encountered detachment of the stent from the balloon during the treatment of BBA, and we discuss the possible reasons for this complication and the way that might avoid it (Table 1). We obtained informed consent for all patients and all patients permitted any form of publication for research purposes.

**Table 1:** Patients, aneurysms and procedures characteristics.

Case	Gender	Aneurysm location	Type of internal carotid artery^a^	Type of detachment	mRS pre-procedure	mRS post-procedure
1	F	C6	IV	Portion	0	0
2	F	C6	II	Whole	2	0

*^a^Reference to Lin et al. (6)*.

## Case Illustration

### Case 1

**Figure 1:**
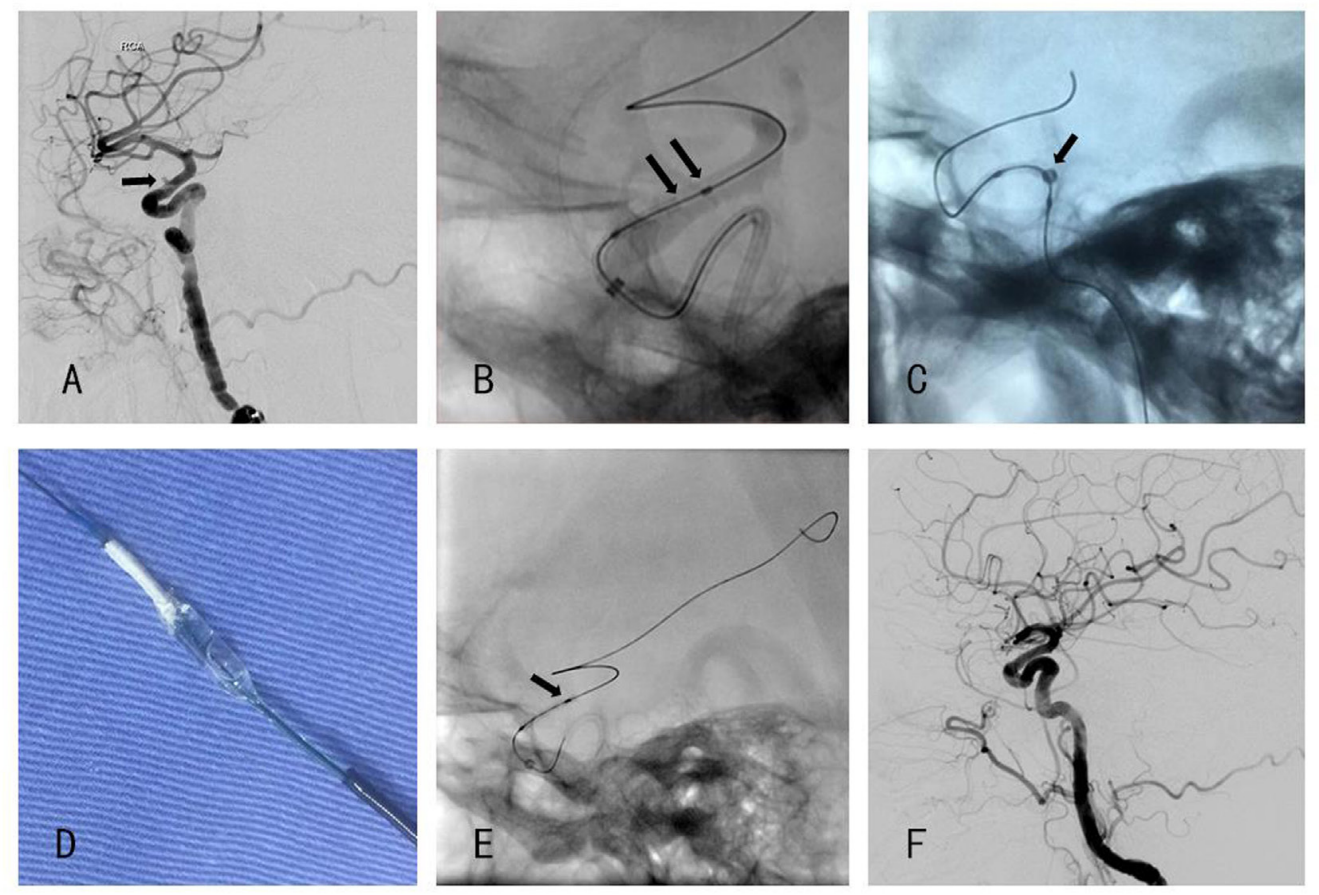
**(A)** Angiography showed a blood blister-like aneurysm. **(B)** The distal end of the stent was detached from the delivery balloon. **(C)** Half-dilated balloon to anchor the stent **(D)** stent anchored to a half-dilated balloon. **(E)** Distal end of the stent was loose when the second Willis covered stent (WCS) was positioned. **(F)** Post-procedural angiography showed that aneurysm was disappeared.

A 52-year-old female with intermittent headache and dizziness was admitted into our department. Computed tomographic angiography showed an aneurysm located at ICA as later verified by the digital subtraction angiography (DSA). The shape and location of the aneurysm indicated the diagnosis of a BBA at C6 segment (ophthalmic segment) of ICA (Figure 1A).

Five days before the procedure, dual antiplatelet therapy (aspirin 100 mg qd and clopidogrel 75 mg qd) was administered. The endovascular procedure was performed under general anesthesia and the right femoral artery was canalized. We adopted a tri-axial system to provide adequate support. That is to place an 8 F guiding catheter (Cordis, Miami, FL, USA) in the C1 segment, and a XT-27 inside the Navien so that the Navien can be carry distally to the aneurysm, thus facilitating the following deployment of the WCS. However, because of the Simmons-style ICA (i.e., posterior genu is buckled superiorly compared with the anterior genu, a shape characteristic of the Simmons-style 2 catheter) and the tortuosity of the petrous segment, the Navien could only be accessed no distal to the C4 segment. Though not satisfying, we proceed the procedure without further introduce the Navien to the supraclinoidal segment. The XT-27 was withdrawn and we used an Echelon-10 and 200-cm synchro-14 to access the M2 segment and then a 300-cm Synchro-14 exchanged the 200-cm microwire. We then chose a WCS measured 3.0 mm × 10.0 mm in size and navigated it over the microwire and bridged the aneurysm neck under roadmap guidance. Before dilation of the balloon, a control DSA was performed to confirm the position, yet we found that the distal end of the stent was already detached from the balloon (Figure 1B). Confronted with this unexpected situation, we got three possible options to choose. Option 1 was to continue dilating the balloon regardless of the danger that the distal end of WCS might further shorten and fail to cover the distal part of the aneurysm neck. Option 2, if the stent did not cover the neck after dilation, we could telescope a second Willis stent distal to the first stent. Though telescoping a WCS was tested in canine model (7) as well as in patients (8), we still regarded telescoping technique in this extremely curved access not feasible since based on our past experience, the microwire most of time failed to pass through the first stent and might be blocked by the proximal strut of the stent. Beside that the friction between the e-PTFE membrane and the microwire is large enough to dislocate the first stent. Option 3 was to retrieve the stent. Finally, the third option was chosen and a half-dilating technique was invented, that is to dilate the balloon at 2–3 atm pressure, thus, the stent was anchored to the balloon while at the same time, the stent was not fully expanded. We then withdrew the delivery wire to make the balloon and the stent corked into the Navien as a whole and eventually, the stent was safely pulled back into the 5 F Navien and retrieved successfully without leaving much damage to the vessel wall as shown by control DSA (Figures 1C,D).

The possible causes of stent detachment might attribute to the tortuous access route. There was a curve at the bifurcation of common carotid artery and one at the lacerum segment of ICA. What is more, the Simmons-style ICA tortuosity would increase intervention failure rate, as reported in the treatment using pipeline embolization device. This vessel configuration is associated with a high rate of mal-apposition of pipeline and insufficient opening of the device (6). Considering that the WCS is more stiff than the Pipeline Embolization Device (PED, Covidien, Irvine, CA, USA), during the advancement of the WCS in this Simmons-style ICA, the friction and torsion were sufficient to make the stent detached. To avoid this complication, choosing a relatively shorter device like a 4.0 mm × 7.0 mm WCS might work, but a WCS sized 7.0 mm increased the difficulty of positioning the device precisely. Based on the abovementioned analysis, we decided to retry a same sized stent, but contrary to the previous procedure, we would navigate the Navien distally to the aneurysm, hoping that this maneuver might provide more access support. As shown in Figure 1E, though not detached, the distal end of the stent was loosened without evident shortening, the Navien was already pushed back to the cavernous segment. We then successfully released the Willis stent (Figure 1F) and the aneurysm was gone. The patient recovered smoothly from the anesthesia without any neurological symptom.

### Case 2

**Figure 2:**
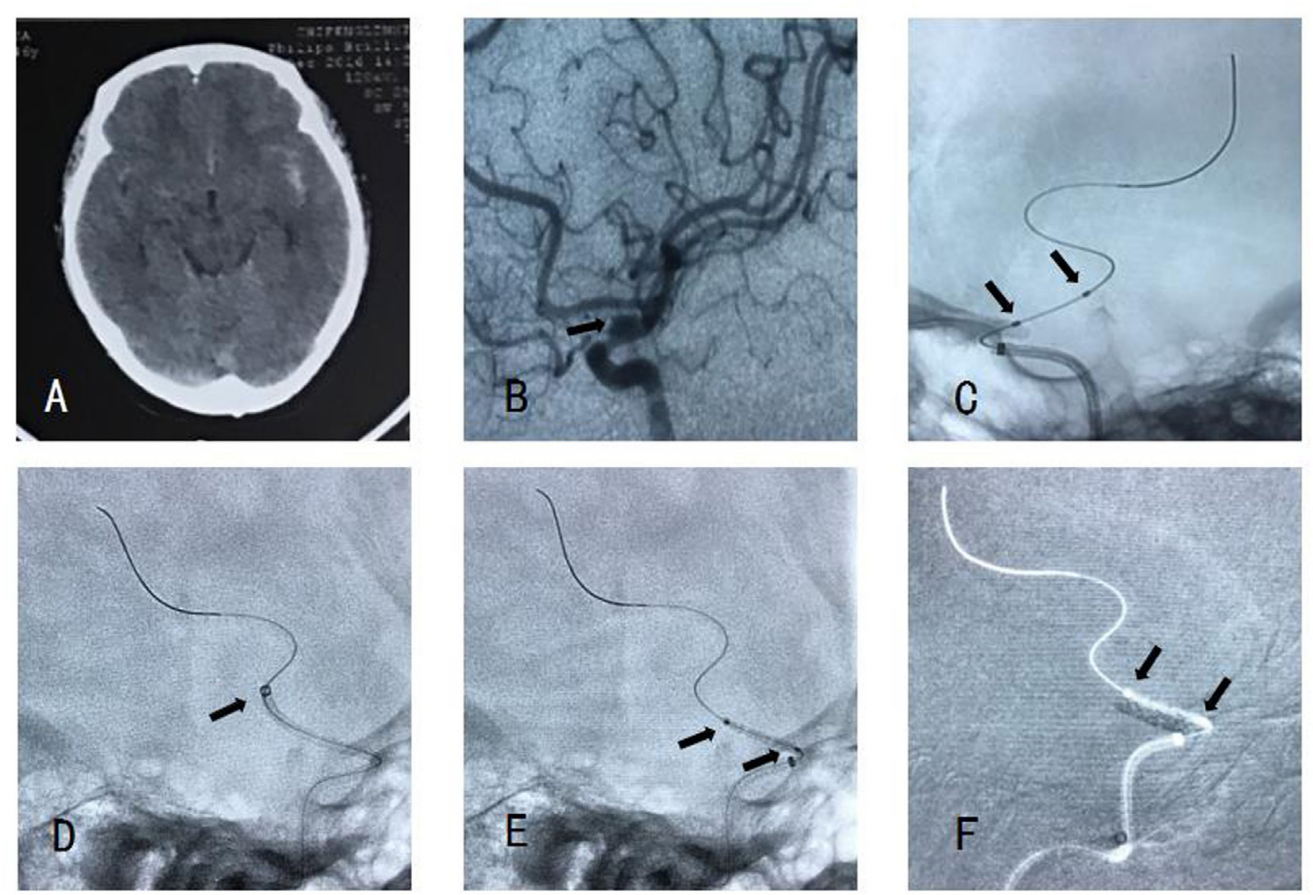
**(A)** Computed tomography showed subarachnoid hemorrhage. **(B)** Angiography showed a blood blister-like aneurysm. **(C)** There is no stent between two markers. **(D)** Navien accessed to distal of aneurysm neck enough. **(E)** Willis covered stent (WCS) accessed to aneurysm neck smoothly. **(F)** The WCS was expanded successfully and covered the aneurysm neck.

A 46-year-old female with sudden severe headache, nausea, and vomiting was admitted into local Hospital. Computed tomography showed subarachnoid hemorrhage (Figure 2A), so she was transferred to our department 3 days later for endovascular treatment. DSA showed a BBA located at C6 segment of ICA (Figure 2B).

Loading dose of dual antiplatelet therapy (300 mg Aspirin and 300 mg Plavix) was administered before procedure. Routine procedure (described previously) was operated, with a type II ICA, the Navien could access to the C5 segment easily, we then navigated a WCS over the microwire under roadmap guidance. When the stent was sent out from Navien, there was no stent between two markers, and we were not sure where the stent was (Figure 2C). We retrieved the microwire and the whole system. In this case, we flushed the microcatheter and then exhaust the gas with angiographic agent extracorporeally before inserting the stent system into the Y-configured valve. This maneuver entailed suction of the balloon from a pump, this will loosen the combining power between the stent and the balloon. So when we enter the stent into the Y valve, the friction between the Y valve and the stent might further loosen the combining force, thus leading premature detachment. So, for this case we chose to navigate Navien to distal of aneurysm neck enough (Figure 2D). We did not exhaust the gas until we navigated WCS to the arcus aortae. Then, the WCS was expanded successfully and covered the aneurysm neck (Figures 2E,F). In our later cases, we intended to further navigate the stent to the cavernous segment of ICA before exhausting the gas in the balloon. So that we could place the WCS and the distal end of the exchanging wire in a zoomed fluoroscopic image. Thus enabled us to detect the move of the distal exchanging wire and eliminated the danger of perforate distal branches. To place the WCS further distal in the Navien or in its final position was not necessary since the WCS would move during gas exhaustion.

## Discussion

Compared with other types of AN, BBA is more dangerous due to their fragile vessel wall and the high-risk to rupture. Numerous treatment methods have been proposed: microsurgical clipping or wrapping, coiling assisted with balloons or stents, and flow diverters. The current literatures have not reached a consensus on an optimal approach for treating this lesion. Though not as frequently reported, WCS is a favored option for BBA in our center considering its ability to reach instant elimination of the aneurysm and durability in follow-up (4).

Here, we reported a complication which we have encountered when BBA was treated with WCS. We believe that three reasons might account for this complication, namely, failed to access the Navien distal to the aneurysm neck in tortuous access route, exhausting the gas extracorporeally, and adjusting the position of the stent repeatedly in the fear of cover the ophthalmic artery (OA) and posterior communicating artery (PcomA). All these three factors augmented the friction between the stent and access route, thus leading to premature detachment. The Navien catheter is relative smooth and flexible, advancing the Navien distally to the aneurysmal neck guaranteed robust support for the stent system and lowered the damage to the vessel caused by the stent navigation, this was true especially in tortuous access route. Exhausting the gas intracorporeally will eliminate an extra loosening of the stent attachment to the balloon. To better detect any dislocation of the distal end of the exchanging wires, we would navigate the WCS to the intracranial portion of ICA in the following operations before gas exhaustion. At last, due to the location of BBA, the aneurysm is distal to the siphon curve and adjacent to OA and PcomA. This special location makes the choice of the stent length difficult. Since a longer stent might have difficulties passing through the siphon curve and cover the orifices of OA and PcomA. A shorter stent may easily across the curve; however, it entails much effort to position the stent properly as to cover the neck of aneurysm without endoleaks. This repeated maneuver might lead to premature detachment. We have not come across such a complication in our earlier Jostent cases since Jostent coronary stent was mainly used to treat lesions proximal to the C5 segment of ICA and rarely been used for classic BBA located in supraclinoidal region. The Simmons-style ICA tortuosity increases the access difficulty of the stent, leaving too much friction and twisting of the device, which ultimately contributes to the detachment.

There are some experiences that should be learnt from these two cases. First, with a tortuous access route, especially when we encounter the Simmons-2-style ICA, we should navigate the Navien distally to the aneurysm neck. Second, intracorporeal gas exhaustion is favored since extracorporeal exhaustion would predispose the stent in a state prone to detach along the access route. Third, try not to repeatedly repositioning the stent in the supraclinoidal segment. In the end, when the stent is partially detached, we can apply the half-dilating technique to retrieve the stent.

## Conclusion

Special cautions should be taken in the face of a Simons-style ICA in the treatment of BBA with a WCS. The tortuosity of the ICA may increase the risk of stent detachment from the delivery wire. To avoid this procedure-related complication, it is feasible to exhaust the gas intracorporeally after navigate the WCS to the intracranial portion of ICA, choose a shorter stent and navigate the Navien distal to the aneurysm. When this complication occurred, the stent can be safely retrieved using the half-dilating technique.

## Ethics Statement

Written informed consent was obtained from the patient for the publication of this case report.

## Author Contributions

YuxiangZ and YupengZ: collected the data, drafted the manuscript, and are assistant performer of the procedures; these two authors are co-first author. FL: collecting the data. CJ: chief performer of procedures and conceived the manuscript.

## Conflict of Interest Statement

The authors declare that the research was conducted in the absence of any commercial or financial relationships that could be construed as a potential conflict of interest.
